# Comparison of hyaluronidase-facilitated subcutaneous immunoglobulin 10% pharmacokinetics in pediatric and adult patients with primary immunodeficiency: an analysis of three phase 3 clinical trials

**DOI:** 10.1093/immadv/ltag022

**Published:** 2026-10-13

**Authors:** Zhaoyang Li, Alex Ballesteros-Perez

**Affiliations:** Clinical Pharmacology & Early Clinical Development, Takeda Development Center Americas, Inc., Cambridge, MA, United States; Clinical Pharmacology, Certara Strategic Consulting, Certara USA, Inc., Radnor, PA, United States

**Keywords:** primary immunodeficiency, hyaluronidase-facilitated subcutaneous immunoglobulin, immunoglobulin G, pediatric patients, pharmacokinetics

## Abstract

**Introduction:**

Facilitated subcutaneous immunoglobulin (fSCIG) 10% is approved for the treatment of primary immunodeficiency (PID) in both pediatric and adult patients. However, it remains unclear whether there is a clinically meaningful difference in the pharmacokinetics (PK) of immunoglobulin G (IgG) between these two populations.

**Methods:**

Serum total IgG concentrations in pediatric and adult patients (aged 3–80 years) with PID from three phase 3 clinical trials of fSCIG 10% were analyzed (NCT00814320, NCT01175213, NCT03277313). Serum total IgG trough concentrations (*C*_trough_) were compared against the conservative threshold for treatment effectiveness of 7 g/l, and between adult and pediatric patients (aged 2 to <17 years) and pediatric subgroups (2 to <12 years and 12 to <17 years). PK parameters, including steady-state maximum concentration (*C*_max_), area under the curve during the dosing interval, normalized by week (AUC/week), and apparent clearance normalized for body weight (CL/F/BW), were also compared across age groups.

**Results:**

All age groups effectively achieved mean IgG *C*_trough_ above 7 g/l following fSCIG 10% administration. Total IgG exposures (mean *C*_trough_, *C*_max_, and AUC/week) marginally increased with age (all *P* < 0.05), while there was considerable variability within age groups. However, after total IgG baseline correction, there were no differences in any exposure parameters by age. CL/F/BW remained consistent across studies and age groups.

**Conclusion:**

fSCIG 10% effectively maintains *C*_trough_ in IgG treatment-experienced patients with PID. The small age-related differences observed in serum total IgG PK are unlikely to be clinically significant and dose adjustments by age alone are not warranted.

## Graphical Abstract

**Graphical Abstract:**
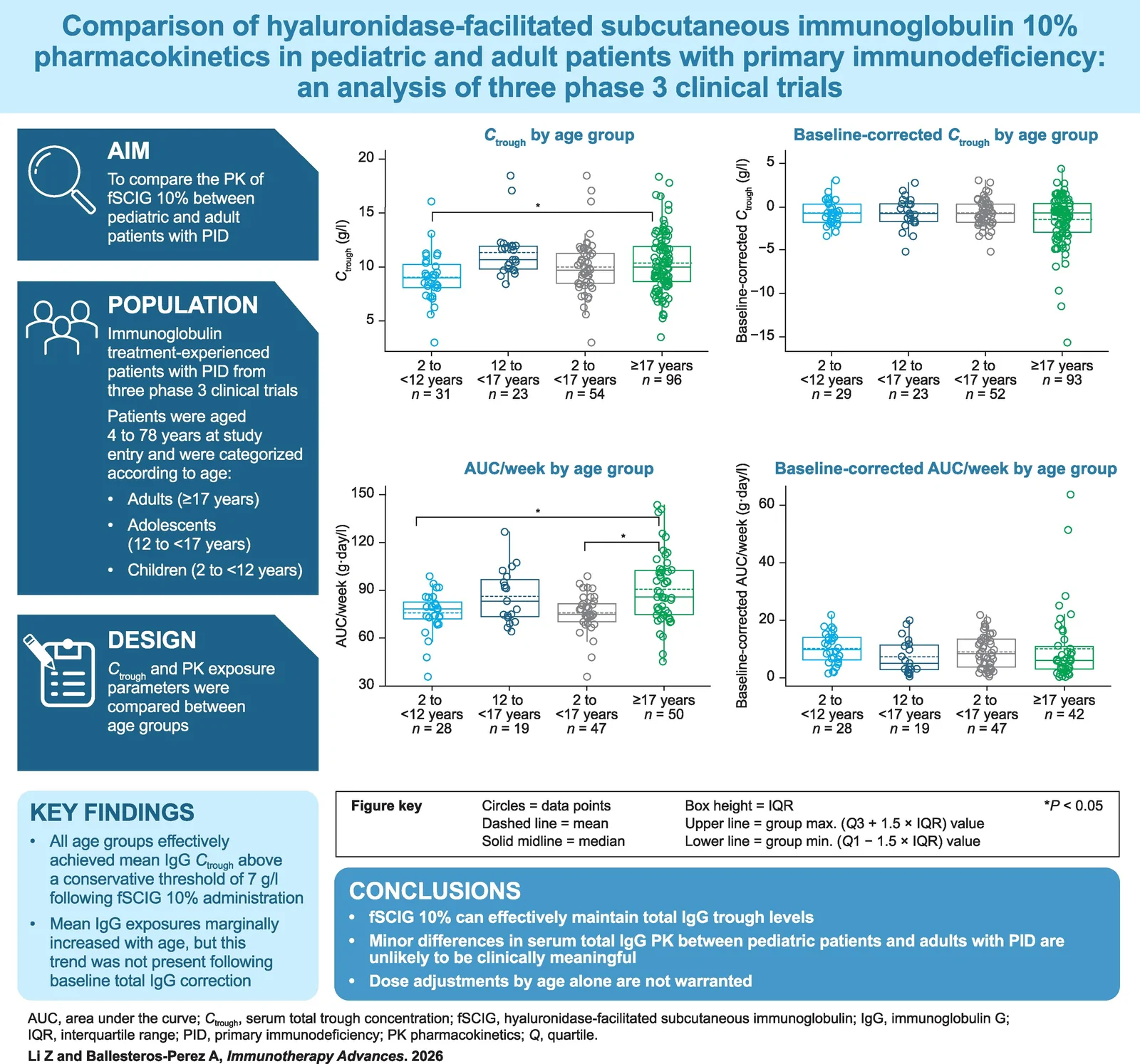

## Introduction

Primary immunodeficiency (PID), also referred to as inborn errors of immunity (IEI), is a group of heterogeneous genetic disorders characterized by a defect in immune system development and/or function [1–4]. Patients with PID have an increased susceptibility not only to infection but also to severe autoinflammation, autoimmunity, allergy, and malignancy [5]. In 2024, the International Union of Immunological Societies Expert Committee updated its classification to include 508 genetic variants resulting in 559 IEI phenotypes [3, 4]. Patients with antibody defects or deficiencies characteristically experience recurring bacterial infections, particularly of the sinopulmonary tract [5–8]. In patients with PID associated with an antibody deficiency, immunoglobulin G (IgG) replacement therapy represents the standard-of-care [2, 9]. Compared with intravenous immunoglobulin (IVIG), conventional subcutaneous immunoglobulin (SCIG) treatment results in fewer systemic adverse events (AEs) and allows self-administration at home [9, 10]. However, because of limitations on infusion volumes per infusion site, SCIG requires more frequent dosing (daily to every 2 weeks) than IVIG (every 3–4 weeks) and may require multiple infusion sites [11–13].

HYQVIA/HyQvia, a hyaluronidase-facilitated subcutaneous immunoglobulin (fSCIG; Baxalta US, Inc., a Takeda company, Cambridge, MA, USA; Baxalta Innovations GmbH, Vienna, Austria), comprises a dual-vial unit of IgG 10% and recombinant human hyaluronidase PH20 (rHuPH20) [14, 15]. rHuPH20 transiently increases subcutaneous tissue permeability to IgG by depolymerizing extracellular hyaluronan, thereby permitting greater IgG dispersion and absorption and allowing for administration of larger volumes and less frequent administration than conventional (without rHuPH20) SCIG [12, 16]. fSCIG 10% is approved for the treatment of PID in patients of all ages in the EU and in patients aged ≥2 years in the USA [14, 15]. fSCIG 10% is also approved for chronic inflammatory demyelinating polyradiculoneuropathy as maintenance therapy after stabilization with IVIG for patients of all ages in the EU and in adults in the USA [14, 15]. In addition, fSCIG 10% is approved for treatment of secondary immunodeficiency disease in patients of all ages in the EU who experience severe recurrent infections, ineffective antimicrobial treatment, and either proven specific antibody failure or a serum IgG level <4 g/l [15]. fSCIG can be a useful treatment option for pediatric patients that can help reduce the burden on daily life. Compared with IVIG, fSCIG offers the opportunity for self-administration at home and is associated with fewer systemic side effects without the need for venous access. In addition, compared with SCIG, fSCIG requires fewer needle sticks, allows for longer infusion intervals and a higher infusion rate [17].

Given the potential variation in drug absorption, distribution, and excretion in patients of different ages [18, 19], it is important to examine whether there are differences in IgG pharmacokinetics (PK) after treatment with fSCIG 10% across age groups and to determine any implications for dosing regimens.

Simulations using a previously published robust population PK model projected slightly lower minimum serum total IgG concentrations in younger age groups for both SCIG and fSCIG 10% [20]. Previous studies also confirmed that all age groups maintained serum total IgG trough concentrations (*C*_trough_) above 7 g/l, which is a more conservative theoretical target concentration above the minimum clinically protective threshold of 5 g/l [20, 21]. To date, there have not been any direct PK comparisons for fSCIG 10% across age groups.

The objective of the current analysis was to compare the PK of IgG in pediatric and adult patients with PID after administration of fSCIG 10% using a large dataset from three prior clinical trials, and to discuss the implications of any potential differences.

## Methods and materials

### Data sources

Data from three phase 3 clinical trials involving immunoglobulin treatment-experienced patients with PID treated with fSCIG 10% were included in this analysis (Table 1). Study 1 (NCT00814320; *N* = 87) was a prospective, open-label, noncontrolled, nonrandomized, multicenter trial in adult and pediatric patients with PID [22]. The trial comprised two epochs: in Epoch 1, participants received IVIG 10% for 3 months at 3- or 4-week treatment intervals; in Epoch 2, participants received fSCIG 10% for 14–18 months at 108% of the weekly equivalent Epoch 1 IVIG dose and the same treatment interval. Study 2 (NCT01175213; *N* = 66) was a long-term extension of Study 1 in which participants continued fSCIG 10% treatment with the option of switching to a shorter 2-week dosing interval [23]. Study 3 (NCT03277313; *N* = 44) was a prospective, open-label, noncontrolled, multicenter trial in pediatric patients in which fSCIG 10% treatment was initiated with a dose ramp-up (Epoch 1) followed by fSCIG 10% administered at 3- to 4-week treatment intervals for 3 years (Epoch 2) [24]. Patient *C*_trough_ were reported in all three studies, and additional PK parameters were available in Studies 1 and 3.

**Table 1:** Clinical trials included in the analysis.

Study number (NCT number)	Study design	Number of patients included in the analysis		Trial objective(s)	Available pharmacokinetic data
		Pediatric (2 to <17 years)	Adult (≥17 years)		
Study 1 (NCT00814320, *n* = 87)	Phase 3, prospective, open-label, noncontrolled, nonrandomized, multicenter trial comprising two epochs Epoch 1: IVIG treatment for 3 months Epoch 2: fSCIG 10% treatment for approximately 14–18 months	18^a^	50^a^	Efficacy, tolerability, quality of life, and pharmacokinetic comparison of IVIG 10% and fSCIG 10%	*C*_trough_ AUC/week, *C*_max_, *T*_max_, *C*_min_, *t*_1/2_, CL/F/BW
Study 2 (NCT01175213, *n* = 66)	Phase 3, prospective, open-label, noncontrolled, multicenter trial; long-term (≤3 years) extension study for patients who had completed NCT00814320	11	46	Long-term tolerability and safety of fSCIG 10%	*C*_trough_
Study 3 (NCT03277313, *n* = 44)	Phase 3, prospective, open-label, noncontrolled, multicenter trial comprising two epochs Epoch 1: fSCIG 10% dose ramp-up Epoch 2: fSCIG 10% treatment for 3 years	44	0	Efficacy, safety assessments, immunogenicity, tolerability, characteristics of product administration, treatment preference and satisfaction, health-related quality of life, and pharmacokinetics relating to fSCIG 10%	*C*_trough_ AUC/week, *C*_max_, *T*_max_, *C*_min_, *C*_trough_, *t*_1/2_, CL/F/BW

^a^Numbers of patients from Epoch 2.

AUC/week, area under the dosing interval curve normalized by week; CL/F/BW, apparent clearance normalized for bodyweight; *C*_max_, maximum concentration; *C*_min_, minimum concentration; *C*_trough_, serum total IgG trough concentration; fSCIG, facilitated subcutaneous immunoglobulin; IgG, immunoglobulin G; IVIG, intravenous immunoglobulin; *t*_1/2_, terminal half-life; *T*_max_, time to *C*_max_.

### PK sample collection

In Study 1, *C*_trough_ were analyzed in blood sampled at the end of Epoch 2 (14–18 months); additional PK parameters were recorded for patients aged ≥12 years from samples taken before the last fSCIG 10% infusion (60 minutes preinfusion and up to 28 days [±2 days] postinfusion). Blood samples for serum total IgG trough analyses in Study 2 were taken at the end of fSCIG 10% infusion cycles throughout the treatment period. In Study 3, *C*_trough_ were recorded in Epoch 2 Month 0 and additional PK parameters were available from samples analyzed after 6 months of fSCIG 10% treatment with 3- or 4-week treatment intervals (Table 1).

### Data handling and analysis

For cross-study comparisons, data were grouped by age according to US Food and Drug Administration clinical pharmacology guidance for pediatric studies: 2 to <12 years for children and 12 to <17 years for adolescents [25]. Patients in Studies 1 and 2 who were aged ≥17 years were considered to be adults in this analysis.

Descriptive summary statistics for age at study entry and *C*_trough_ were calculated for each age group. Descriptive statistics were summarized for the following additional serum total IgG PK parameters common to both Study 1 and Study 3: steady-state total IgG exposure (area under the dosing interval curve normalized by week [AUC/week]), minimum concentration (*C*_min_), maximum concentration (*C*_max_), time to *C*_max_ (*T*_max_), terminal half-life (*t*_1/2_), and apparent clearance normalized for body weight (CL/F/BW). Baseline-corrected serum total IgG trough and serum total IgG PK parameters were calculated. Differences in total IgG PK parameters across age groups were compared with one-way ANOVA and Dunnett’s post hoc analysis using the adult group in Study 1 as the reference. Statistical significance was defined as *P* < 0.05.

PK parameters were obtained by noncompartmental analysis using Phoenix WinNonlin (Certara; Princeton, NJ, USA; version 8.1.0), and data summaries and graphical outputs were produced using R (R Core Team; open-source; version 4.2.2) and Phoenix WinNonlin.

This analysis utilized de-identified aggregated existing data from previous clinical trials; therefore, no further ethical approval was required or sought. The original studies were approved by the appropriate ethics committees in line with relevant guidelines.

## Results

### Patient characteristics

At study entry, the ages of patients included in this analysis ranged from 4 to 78 years (*n* = 68) in Study 1 and from 9 to 80 years (*n* = 57) in the long-term extension (Study 2); in Study 3, pediatric patients were aged 3–15 years (*n* = 44). The median age within the age categories used for this analysis was similar across studies (Table 2).

**Table 2:** Descriptive statistics for age at study entry and *C*_trough_ after fSCIG 10% treatment.

Parameter	Age group			
	Children (2 to <12 years)	Adolescents (12 to <17 years)	All pediatric patients (2 to <17 years)	Adults (≥17 years)
**Age at study entry, years**				
Study 1 (NCT00814320)				
*n*	8	10	18	50
Median (range)	8.0 (4–11)	14.0 (12–16)	12.0 (4–16)	52.5 (17–78)
Study 2 (NCT01175213)				
*n*	4	7	11	46
Median (range)	10.5 (9–11)	15.0 (13–15)	13.0 (9–15)	53.5 (17–80)
Study 3 (NCT03277313)				
*n*	32	12	44	0
Median (range)	7.0 (3–11)	13.5 (12–15)	9.5 (3–15)	NC
***C*_trough_, g/l**				
Study 1 (NCT00814320)^a^				
*n*	0	10	10	50
Mean (SD)	NC	11.9 (2.65)	11.9 (2.65)	10.5 (2.69)
Study 2 (NCT01175213)^b^				
*n*	4	7	11	46
Mean (SD)	10.1 (4.02)	11.7 (2.59)	11.1 (3.08)	10.3 (2.83)
Study 3 (NCT03277313)^c^				
*n*	27	6	33	0
Mean (SD)	8.9 (2.05)	10.1 (0.51)	9.1 (1.91)	NC

^a^After 14–18 months of fSCIG 10% treatment with 3- or 4-week treatment intervals.

^b^After fSCIG 10% treatment with 4-week treatment intervals.

^c^During Epoch 2, Month 0.

Pediatric patient data for Studies 1 and 2 were assigned to the age groups used in Study 3, with an additional adult age group (≥17 years).

*C*_trough_, serum total IgG trough concentration; fSCIG, facilitated subcutaneous immunoglobulin; IgG, immunoglobulin; NC, not calculated; SD, standard deviation.

The most common PID diagnosis in the overall Study 1 population was common variable immunodeficiency (CVID; 49/87 patients [56%]), followed by hypogammaglobulinemia (17/87 patients [20%]) [22]. In Study 3, CVID was the most common diagnosis (18/44 patients [41%]), followed by specific antibody deficiency (16/44 patients [36%]) and hypogammaglobulinemia (8/44 patients [18%], including four patients with specific antibody deficiency) [24].

### Cross-study analyses of PK parameters by age

Across studies, all age groups achieved mean serum total IgG *C*_trough_ above a conservative threshold of 7 g/l after fSCIG 10% administration (Table 2). Without total IgG baseline correction, there was a slight trend of *C*_trough_ values increasing with age, with mean *C*_trough_ (standard deviation [SD]) ranging from 8.9 (2.0) g/l to 11.9 (2.7) g/l across age groups and across studies (Fig. 1a). Observed differences were not statistically significant, except for mean *C*_trough_ values between adults and pediatric patients aged 2 to <12 years (*P* < 0.05; Fig. 1a). Baseline-corrected *C*_trough_ values did not differ across age groups (Fig. 1b).

**Figure 1:**
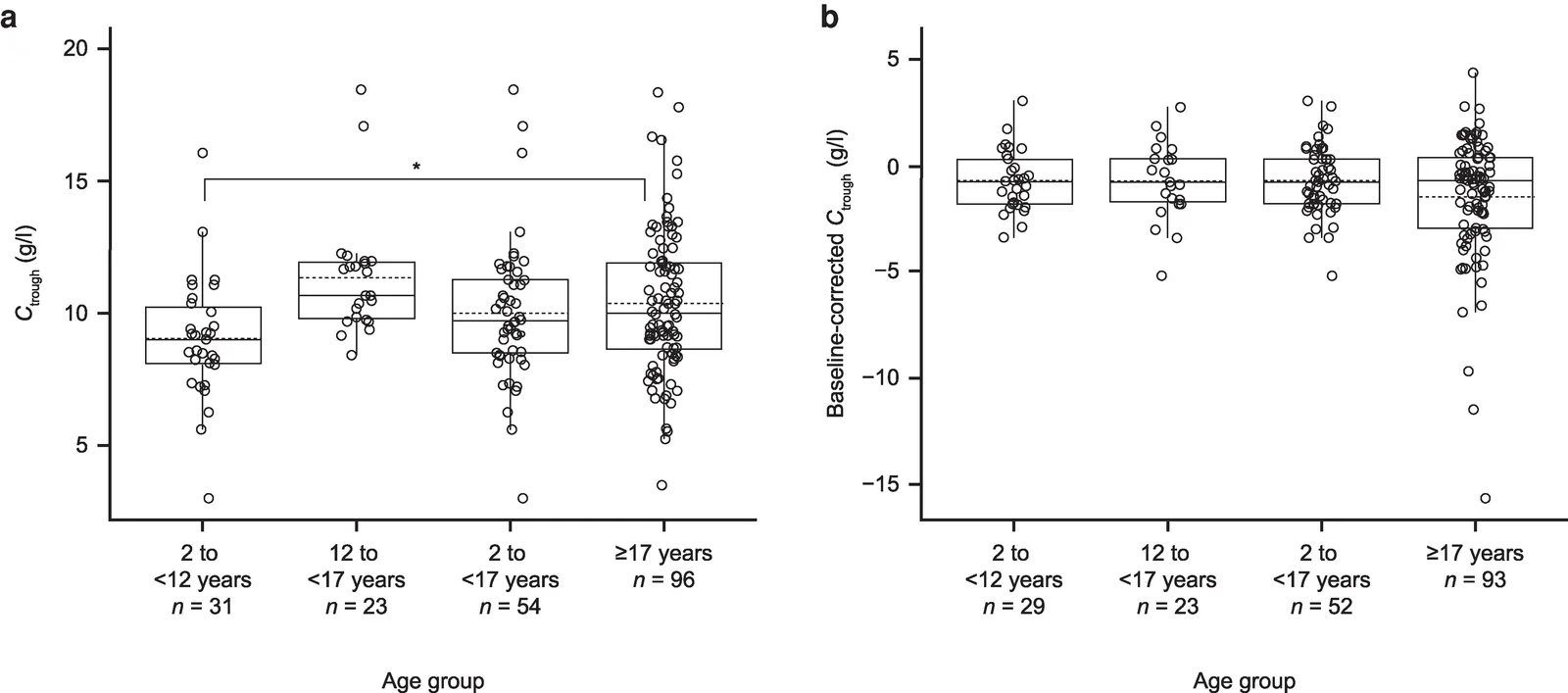
Comparison of serum IgG: (a) *C*_trough_ and (b) baseline-corrected *C*_trough_ across age groups. Boxplots of steady-state serum IgG *C*_trough_ levels in pediatric patients from studies 1, 2, and 3 compared with adult patients from studies 1 and 2. *Statistically significant at *P* < 0.05. Age groups were based on patient age at screening. Solid horizontal lines in the boxes represent group medians; dashed lines indicate means; box heights represent IQRs; upper and lower error bars represent group maximum (*Q*3 + 1.5 × IQR) and minimum (*Q*1–1.5 × IQR) values, respectively; black circles represent observed values. *C*_trough_, serum total IgG trough concentration; IgG, immunoglobulin G; IQR, interquartile range; *Q*, quartile.

Descriptive statistics for PK parameters after fSCIG 10% treatment in Studies 1 and 3 are shown across age groups in Table 3. There was considerable variability within age groups and between studies, and higher mean and median exposure parameters (*C*_max_, AUC/week and *C*_min_) in patients aged 12 to <17 years in Study 1 versus the same age group in Study 3 were reported. This could be at least partially attributed to the higher dose of fSCIG 10% in Study 1 when 108% dosing conversion was used for patients switching from IVIG to fSCIG 10%. Mean (SD) *C*_max_ values were similar between the age groups within each study, with values in Study 1 of 16.6 (3.4) g/l in the 12 to <17 years age group and 16.0 (3.9) g/l in the adult group; corresponding data in Study 3 were 13.2 (2.1) g/l in the 2 to <12 years age group and 12.6 (1.5) g/l in the 12 to <17 years group. The difference in *C*_max_ between adults and all pediatric patients from Studies 1 and 3 combined (aged 2 to <17 years) was statistically significant, as was the difference between adults and pediatric patients aged 2 to <12 years (both *P* < 0.05; Fig. 2a). There were no statistically significant differences in baseline-corrected *C*_max_ values between age groups (Fig. 2b).

**Figure 2:**
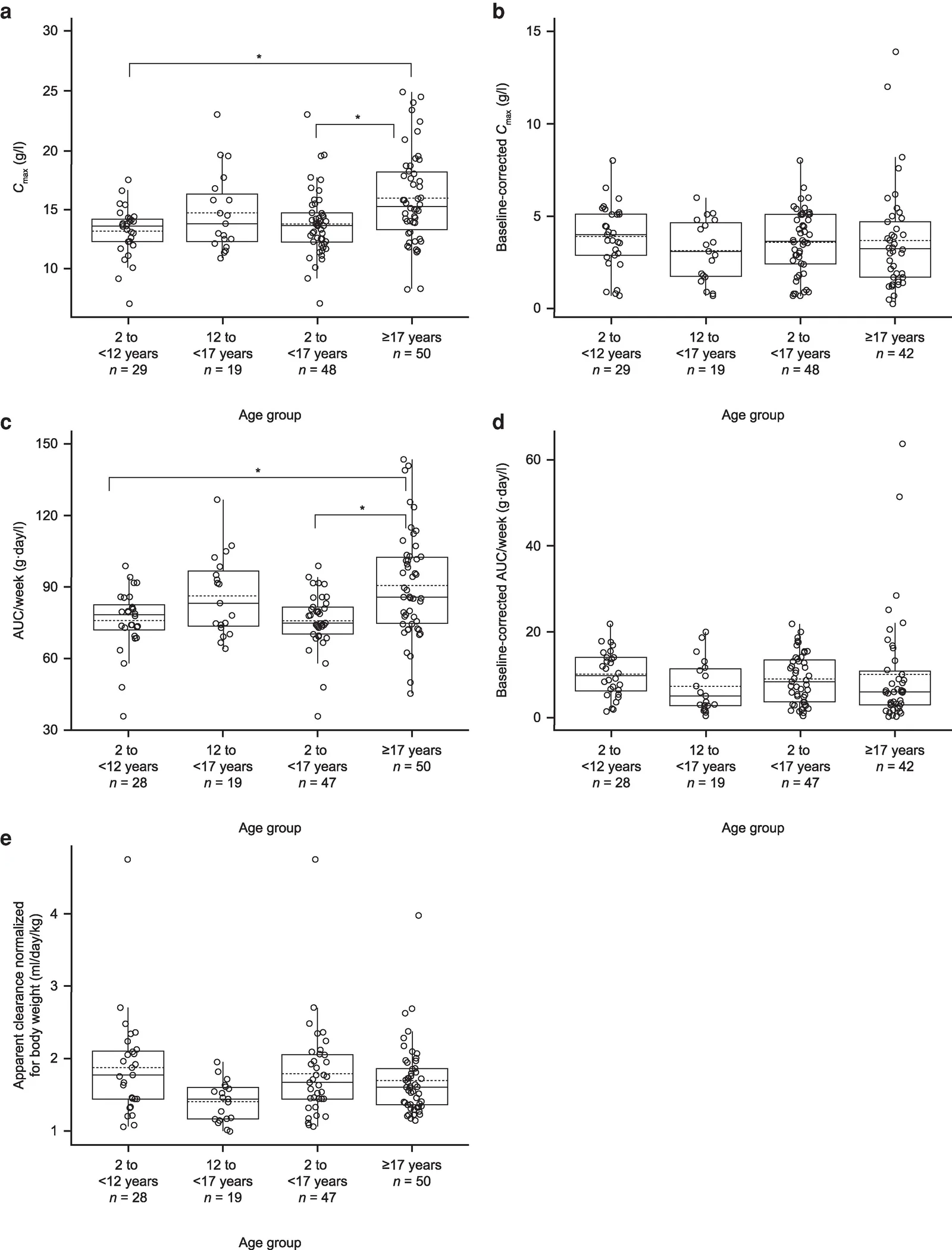
Comparison by age group of total IgG: (a) *C*_max_, (b) baseline-corrected *C*_max_, (c) AUC/week, (d) baseline-corrected AUC/week, and (e) apparent clearance. Boxplots of steady-state serum total IgG levels in pediatric patients from studies 1 and 3 compared with adult patients from study 1. *Statistically significant at *P* < 0.05. Age groups were based on patient age at screening. Solid horizontal lines in the boxes represent group medians; dashed lines indicate means; box heights represent IQRs; upper and lower error bars represent group maximum (*Q*3 + 1.5 × IQR) and minimum (*Q*1–1.5 × IQR) values, respectively; black circles represent observed values. AUC, area under the concentration–time curve; *C*_max_, maximum serum total IgG concentration; IgG, immunoglobulin G; IQR, interquartile range; *Q*, quartile.

**Table 3:** Pharmacokinetic parameters for serum total IgG after treatment with fSCIG 10% in pediatric patients and adults.

	Age group (years)				
	Study 3 (NCT03277313)			Study 1 (NCT00814320)	
Parameter	Children (2 to <12 years) *n* = 29	Adolescents (12 to <17 years) *n* = 9	All pediatric patients (2 to <17 years) *n* = 38	Adolescents (12 to <17 years) *n* = 10	Adults (≥17 years) *n* = 50
AUC/week, g**·**day/l Mean (SD) Median (range)	75.7 (13.4)^a^ 78.2 (35.4–98.7)	75.6 (7.6) 74.5 (66.4–91.1)	75.7 (12.2)^b^ 74.7 (35.4–98.7)	95.6 (17.6) 96.7 (63.8–126.7)	90.5 (21.7) 85.7 (45.0–143.7)
CL/F/BW, ml/day/kg Mean (SD) Median (range)	1.88 (0.7)^a^ 1.8 (1.1–4.8)	1.52 (0.3) 1.5 (1.1–2.0)	1.79 (0.6)^b^ 1.7 (1.1–4.8)	1.30 (0.3) 1.2 (1.0–1.8)	1.69 (0.5) 1.6 (1.1–4.0)
*C*_max_, g/l; mean (SD)	13.2 (2.1)	12.6 (1.5)	13.0 (2.0)	16.6 (3.4)	16.0 (3.9)
*C*_min_, g/l; mean (SD)	8.81 (2.0)	8.76 (1.2)	8.80 (1.8)	11.1 (2.1)	10.2 (2.7)
*T*_max_, day; median (range)	5.1 (2.1–26.9)	4.1 (2.0–7.8)	5.0 (2.0–26.9)	3.1 (2.6–13.8)	5.0 (1.2–15.0)
*t*_1/2_, day; mean (SD)	52.0 (29.6)^c^	39.2 (8.3)^d^	49.8 (27.3)^e^	83.5 (56.2)	54.5 (29.0)

^a^ *n* = 28.

^b^ *n* = 37.

^c^ *n* = 14.

^d^ *n* = 3.

^e^ *n* = 17.

AUC/week, area under the dosing interval curve, normalized by week; *C*_max_, maximum serum total IgG concentration; *C*_min_, minimum serum total IgG concentration; CL/F/BW; apparent clearance, normalized for body weight; fSCIG, facilitated subcutaneous immunoglobulin; IgG; immunoglobulin G; SD, standard deviation; *t*_1/2_, terminal half-time; *T*_max_, time to maximum concentration.

In Study 1, mean (SD) total IgG AUC/week was 95.6 (17.6) g**·**day/l in the 12 to <17 years age group and 90.5 (21.7) g**·**day/l for adults. In Study 3, mean (SD) AUC/week was 75.7 (13.4) g**·**day/l in the 2 to <12 years age group, and 75.6 (7.6) g**·**day/l for the 12 to <17 years age group (Table 3). There were statistically significant differences in mean AUC/week values between the adult group in Study 1 and pediatric patients overall (aged 2 to <17 years) from Studies 1 and 3, and between adults and those aged 2 to <12 years in Study 3 (both *P* < 0.05; Fig. 2c). After total IgG baseline correction, there were no statistically significant differences in baseline-corrected AUC/week values between age groups (Fig. 2d).

There were no statistically significant differences between groups in apparent clearance (Fig. 2e). Apparent clearance ranged from a mean (SD) of 1.3 (0.3) mL/day/kg to 1.9 (0.7) mL/day/kg across studies and across age groups (Table 3).

## Discussion

Serum total IgG PK—particularly trough levels—have long served as a surrogate marker for the efficacy of immunoglobulin therapy and remain central to the treatment paradigm for PID [16, 26–29]. Although dosing levels are traditionally based on body weight, questions have been raised about whether developmental changes in body composition might necessitate age-specific adjustments [12, 20].

This cross-study comparison was conducted to evaluate the PK of serum total IgG after administration of fSCIG 10% in immunoglobulin treatment-experienced adult and pediatric patients with PID. Given the previously reported differences in drug exposure between adults and pediatric patients [18, 19], particularly in the youngest age groups, it was important to conduct a comprehensive assessment of serum IgG PK by age to elucidate any implications for dosing. Across three phase 3 trials, *C*_trough_ were largely consistent across pediatric age groups with marginal elevations observed with increasing age—a trend also seen in healthy children [30]. Results of the current study are also consistent with those of previous simulations of fSCIG dosing every 4 weeks in immunoglobulin treatment-experienced patients (including data from Studies 1 and 3), which showed a trend for lower minimum serum total IgG concentrations across decreasing age groups (≥18, 12 to <18, 6 to <12, and 2 to <6 years) [20]. In the current study, small but statistically significant differences were observed in exposure parameters (*C*_max_ and AUC/week) between adults and the pediatric groups combined in Studies 1 and 3; however, the variability of the adult exposures was wide, encompassing almost all data points reported within the pediatric groups. Additionally, there were no differences in total IgG baseline-corrected exposure parameters. Taken all together, these differences between pediatric patients and adults are unlikely to represent a clinically meaningful difference. Apparent clearance normalized for body weight showed no clear difference between pediatric patients and adults. During the development of a population PK model integrating data from clinical trials of IVIG, conventional SCIG, and fSCIG in PID, there was a trend for lower IgG clearance with greater age but no statistically significant covariate effect of age on IgG clearance [31], which was consistent with the findings of another modeling investigation of IVIG in adults and children with PID [32].

Notably, across all studies included in the current analysis, all age groups achieved mean *C*_trough_ above 7 g/l, which is a conservative theoretical target above the commonly accepted minimum threshold of 5 g/l considered necessary to reduce the risk of infection in patients with PID and hypogammaglobulinemia [21]. The lowest mean *C*_trough_ recorded among all age groups across the three studies were 8.9 g/l in the 2 to <12 years age group in Study 3. Overall infection rates were low among fSCIG 10%-treated participants in the studies included in this analysis [22–24]. For example, in Study 1, the overall rate of infections across adults and pediatric patients was 2.97 per patient-year (95% confidence interval [CI] 2.51–3.47) [22]. Infection rates per patient-year were similar between those aged <18 years (3.02, 95% CI 2.15–4.10) and those aged ≥18 years (2.98, 95% CI 2.56–3.44) in Study 2 [23]. The rates of acute serious bacterial infections (ASBIs) per patient-year were low in both adult and pediatric cohorts (0.08 and 0.01 ASBIs per year, respectively) in Study 2 [23] and 0.04 per patient-year for pediatric patients in Study 3 [24]. These efficacy findings, in addition to *C*_trough_ remaining above 7 g/l in both adult and pediatric groups, support fSCIG 10% as an effective maintenance therapy for patients at least 2 years of age.

Children with PID in particular may benefit from conventional SCIG or fSCIG owing to difficulties with venous access [33]. Additionally, unlike IVIG, conventional SCIG and fSCIG infusions can be administered at home, improving quality of life and providing greater control for daily living [10, 34]. However, a limitation of conventional SCIG is the limited volume of IgG that can be administered at a single site or a single infusion, necessitating more frequent infusions than IVIG [11–13]. fSCIG overcomes this limitation by enabling higher volumes to be administered in a single infusion, thereby combining the benefits of both IVIG and conventional SCIG [9, 12, 35, 36].

Given that this retrospective analysis is a cross-study comparison, differences in methods between the studies cannot be totally excluded. Additionally, there were slight differences in the diagnoses of PID between the studies’ populations, with a greater proportion of patients in Study 1 with CVID than in Study 3, and a greater proportion of patients in Study 3 with specific antibody deficiency than in Study 1, which could result in differing responses to IgG treatment. Relatively small sample sizes are a common limitation of PK analyses of pediatric patient data from clinical trials due to the difficulties with the number and volume of blood samples that can be obtained from children [37].

In summary, this cross-study comparative analysis of adult and pediatric patient data from clinical trials of fSCIG 10% in treatment-experienced patients with PID demonstrated that fSCIG 10% was able to effectively maintain *C*_trough_ above the clinically therapeutic target in all age groups. There was a slight tendency of increased exposure with rising age, while wide inter-patient variability was observed. After normalization for body weight, apparent clearance in pediatric patients and adults did not differ. The differences in total IgG concentrations between pediatric and adult patients, although statistically significant, were small and unlikely to be clinically meaningful. Additionally, those observed differences between pediatric and adult patients diminished after IgG baseline correction, further confirming the comparability of total IgG PK across age groups.

Overall, our findings demonstrate that age-related differences in IgG PK are minimal, supporting the similar use of individualized dosing strategies for fSCIG 10% in pediatric patients, as is done in adults. These results indicate that age-based dose adjustments are not warranted; rather, dosing should be guided by the physician’s clinical judgment, informed by the patient’s IgG trough levels and individual therapeutic needs.

## Data Availability

The data sets will be made available within 3 months of initial request to researchers who provide a methodologically sound proposal. The data will be provided after its de-identification, in compliance with applicable privacy laws, data protection, and requirements for consent and anonymization.
